# Conservation Biogeography: The Paradigm Shift Needed to Reform the Conservation Spatial Planning Sector

**DOI:** 10.1002/ece3.73564

**Published:** 2026-04-20

**Authors:** Clinton Carbutt

**Affiliations:** ^1^ School of Agriculture and Science University of KwaZulu‐Natal Scottsville South Africa; ^2^ Scientific Services Ezemvelo KZN Wildlife Cascades South Africa

**Keywords:** administrative planning domain, biogeographic ecoregions, endemism, systematic conservation planning

## Abstract

Conservation biogeography is increasingly important in biodiversity conservation, but its potential has not been fully integrated into the conservation spatial planning sector. These planning mandates are often carried by government agencies bound by regional administrative and legislative frameworks, resulting in planning domains poorly aligned with biogeographic boundaries. Selection of endemic taxa as input data in systematic conservation planning (SCP) should be drawn from published centres of endemism rather than a homogenized selection drawn from expansive administrative boundaries misaligned with natural evolutionary boundaries. Furthermore, given the constrained budgets in conservation governance, it seems plausible to use SCP for the identification of areas critical to the persistence of range‐restricted, congruent assemblages of plants and animals. This approach could optimize the selection and protection of tightly coupled plants and animals within a framework cognizant of the impacts of human‐induced threats. Conservation biogeography is the paradigm shift needed to reform the conservation spatial planning sector.

## 1 Introduction

Biodiversity conservation has historically focused on species (Rebelo and Tansley 1993; D'Antraccoli et al. 2023), ecosystems (Hjerpe et al. 2015) through to global biomes (Carbutt et al. 2017). To better protect representative samples of biodiversity in optimal areas, spatial biodiversity conservation has embraced tools such as systematic conservation planning (SCP) to identify and potentially secure priority areas with the lowest opportunity costs (Margules and Pressey 2000). SCP is constantly evolving and progressing through new approaches that incorporate novel criteria to identify spatial priorities. Examples include climate change adaptation (Groves et al. 2012), environmental gradients (Jewitt et al. 2015), species' traits—the cornerstone of functional diversity (Trethowan et al. 2025), and deeper signals deciphered through evolutionary history (phylogenetic diversity) (Buerki et al. 2015). These more robust approaches are sensitive to the growing needs of species and ecosystems. SCP has not, however, sufficiently bridged the divide into biogeography. Misalignment with, or failure to explicitly integrate biodiversity from discrete biogeographic realms, regions, ecoregions and centres, has resulted in some spatial plans lacking cohesion and scientific integrity. Conservation biogeography framed at meaningful spatial scales, taking cognisance of the impact of human‐induced changes and threats, is especially important in the dispensation of global change.

## 2 An Operational Perspective

Securing the few remaining large natural areas is the single most important area‐based intervention for the Convention on Biological Diversity's 30 × 30 campaign (Riva et al. 2024). Identifying where these areas are, and the mechanisms required to ensure their protection, are critical steps for biodiversity conservation. Such choices and decision making around how protection is coordinated have the largest impact on success (Eckert et al. 2023). A key foundational step in this process is the production of rigorous spatial plans. Often, the planning domains are aligned with administrative boundaries such as countries, states, provinces, or other regions (Groves et al. 2012; Kraus et al. 2023). The reason is because the spatial planning mandate is vested with national or provincial (state) government conservation agencies bound by administrative and legal frameworks. Such delineations are not always biologically meaningful and may fail to capture natural or coherent patterns of distribution and how they may change over space and time.

Endemism is highly important for the prioritization of conservation efforts (Kier et al. 2009). In biodiverse South Africa, for example, government scientists use endemic species' distributions as part of the spatial planning process. The endemism data layers, organized by taxonomic group, are based on species confined to a provincial boundary as the planning domain. But is this homogenized regional approach scientifically meaningful? Undertaken intentionally or not, the adverse consequences of not explicitly integrating biogeographical frameworks include a less robust spatial plan that may overlook irreplaceable ancient lineages potentially central to ecosystem stability and resilience, or the best at adapting to climate change. Instead, a conservation biogeographic approach would look fundamentally different. A brief plant‐based example is provided as proof‐of‐concept for one of South Africa's most biodiverse provinces, KwaZulu‐Natal, whose biodiversity is managed by a regional conservation authority. This province is represented by four Centres of Floristic Endemism (CFE), although none of which are limited exclusively to KwaZulu‐Natal. From west to east, these are as follows: the Drakensberg Mountain Centre (Carbutt 2019); Greater Midlands Centre (Carbutt 2023), Pondoland Centre (Van Wyk and Smith 2001), and the Maputaland Centre (Van Wyk and Smith 2001). Applying the SCP principles of representation and persistence, it is more intuitive and biogeographically meaningful to select a representative subset of plant endemics with the highest threat status from each CFE as a contribution to the final product. Without abdicating regional administrative mandates, there is scope to first develop an intermediary spatial layer based on the subdivision of a politically defined province into biogeographic (biotic) provinces and ecoregions. Specifically, from an operational perspective, species data files that feed into the spatial planning software should first be represented by subsets of taxa from all CFE rather than a single file representing the homogenous, politically defined planning domain. Although using a terrestrial plant example on a provincial scale, this principle could extend to animal taxa and to other spatial scales linked to larger administrative planning domains.

More than academic, these selections draw from published CFE, each of which represents a shared evolutionary history carved out in a stable climatic refugium (Harrison and Noss 2017). This discrete centre‐by‐centre approach, sensitive to its biogeographic context, would lead to improved conservation outcomes. Some examples include maximizing phylogenetic diversity (PD), which is lost at higher rates than species diversity (Buerki et al. 2015). This is important because these areas may harbor ancient, evolutionary unique lineages with high functional diversity. This may safeguard diverse traits and future adaptability. This approach will also maximize our understanding of other evolutionary processes like speciation, extinction, dispersal, and adaptive radiation central to biogeography (Wen et al. 2013), and better represent the conflating influences of environmental gradients shaped by climate, latitude, elevation, and geology (Jewitt et al. 2015). Collectively, integration of these sum‐of‐parts CFE is therefore more representative of a climatically, topographically and floristically diverse province. This will result in a spatial product better aligned with, and more sensitive to, natural evolutionary boundaries that hardwire the value of stenotopic species that evolved in relative isolation into spatial plans. Ultimately, biodiversity‐informed approaches based on the most meaningful choices will lead to greatest protection (Eckert et al. 2023).

Taking this further, although SCP integrates endemic species into spatial plans, it does not produce explicit biogeographic maps of centres of endemism. Rather, spatial plans based on SCP apply irreplaceability and complementarity analyses using a wider range of features, processes and design criteria (see Smith and Leader‐Williams 2006). Perhaps there is scope to use SCP or an equivalent approach more directly to map CFE and the zoogeographical equivalents rather than focusing on predictive species distribution modeling. Given the high operating costs and constrained budgets associated with conservation governance, a rapid and more integrated approach is needed to capture the “best of the best” at the lowest opportunity costs. It may therefore be feasible in future to combine centres of plant and animal endemism into biological centres of endemism (BCE). This is additionally important given the absence of universal criteria for defining centres and regions of endemism.

This is achieved by identifying fine‐scale hotspots with high congruent animal and plant endemic richness, to identify the most actionable priority conservation areas with the highest combined biological value. This is particularly important since historical protected area networks align poorly with concentrations of endemics (Carbutt 2023; Eckert et al. 2023), rendering such species vulnerable to habitat loss and overutilization. This equally weighted spatial optimization of BCE is critical to the persistence of discrete, range‐restricted assemblages of plants and animals at a fine spatial scale even if the assembly rules are sometimes different for accumulating endemic richness across taxonomic groups. BCE are especially relevant to tightly coupled plant and animal assemblages such as plant–pollinator networks codependent for their survival through co‐evolution and adaptive radiations. The nearest equivalents are Global Biodiversity Hotspots (Myers et al. 2000) but are unfortunately too broad, lacking the spatial resolution to be meaningful at local scales and strictly speaking have a floristic bias according to their defining criteria.

## 3 Conclusion

The use of politically defined planning domains often reflects administrative and legislative mandates, and not necessarily a lack of scientific awareness of biogeographic patterns. Therefore, the key unresolved challenge may be less about recognizing the need for biogeographically meaningful planning units, and more about understanding what constrains the commissioning and implementation of SCP at those scales. Addressing this issue requires engagement with scientific, governance and policy structures that extend beyond the scope of conservation biogeography alone. These are complex matters not easily or quickly resolved.

Notwithstanding the above, there are limitations associated with the use of administratively defined planning domains in conservation spatial planning. Conservation biogeography is the paradigm shift needed to reform the conservation spatial planning sector, particularly in a dispensation of global change characterized by human‐induced impacts and threats. Future spatial plans should be more biogeographically meaningful by embracing robust biogeographic frameworks within which to select representative taxa for modeling and hardwiring into spatial plans.

## Author Contributions

**Clinton Carbutt:** conceptualization (equal).

## Funding

The author has nothing to report.

## Conflicts of Interest

The author declares no conflicts of interest.

## Data Availability

The author has nothing to report.
